# Hydraulic calcium silicate-based root canal sealers mitigate proinflammatory cytokine synthesis and promote osteogenesis *in vitro*

**DOI:** 10.1016/j.jds.2022.12.019

**Published:** 2023-01-07

**Authors:** Aseel Alchawoosh, Kentaro Hashimoto, Nobuyuki Kawashima, Sonoko Noda, Kosuke Nozaki, Takashi Okiji

**Affiliations:** a Department of Pulp Biology and Endodontics, Division of Oral Health Sciences, Graduate School of Medical and Dental Sciences, Tokyo Medical and Dental University, Tokyo, Japan; b Department of Advanced Prosthodontics, Division of Oral Health Sciences, Graduate School of Medical and Dental Sciences, Tokyo Medical and Dental University, Tokyo, Japan

**Keywords:** Cytokine, Differentiation, Endodontic obturation, Macrophage, Osteoblast, Calcium signaling

## Abstract

**Background/purpose:**

The mineralized tissue-inductive ability and anti-inflammatory properties of hydraulic calcium silicate-based (HCSB) sealers have not been fully elucidated. This study aimed to evaluate the effects of the HCSB sealers Bio-C sealer (BioC), Well-Root ST (WST), and EndoSequence BC sealer (BC), on osteoblastic differentiation/mineralization and proinflammatory cytokine synthesis by macrophages.

**Materials and methods:**

Diluted extracts of set sealers or calcium chloride solutions of approximately equivalent Ca^2+^ concentrations were applied to a mouse osteoblastic cell line (Kusa-A1 cells) and lipopolysaccharide-stimulated mouse macrophage cell line (RAW264.7 cells). Expressions of osteoblastic markers in Kusa-A1 cells and proinflammatory cytokines in RAW264.7 cells were evaluated by reverse transcription-quantitative polymerase chain reaction and enzyme-linked immunosorbent assays. Mineralized nodules were detected by Alizarin red S staining. Cell proliferation was assessed by WST-8 assay and cell attachment on set sealers was examined by scanning electron microscopy.

**Results:**

The three sealer extracts significantly upregulated osteocalcin and osteopontin mRNA, and promoted significant mineralized nodule formation in Kusa-A1 cells. The three sealer extracts significantly downregulated the mRNA expressions of interleukin (IL)-1α, IL-1β, IL-6, and tumor necrosis factor (TNF)-α and protein levels of IL-6 and TNF-α in RAW264.7 cells. Calcium chloride solutions induced osteoblastic differentiation/mineralization. AH Plus Jet (a control sealer) extract did not. The three HCSB sealers did not interfere with the growth and attachment of Kusa-A1 cells.

**Conclusion:**

BioC, WST, and BC were biocompatible, upregulated osteoblastic differentiation/mineralization, and downregulated proinflammatory cytokine expression. Ca^2+^ released from HCSB sealers might be involved, at least in part, in the induction of osteoblastic differentiation/mineralization.

## Introduction

The primary objective of endodontic treatment is to eliminate intracanal bacteria mechanically and chemically, which is followed by complete obturation of the root canal space with root canal filling materials.^1^^,^^2^ The essential biological property of root canal filling materials is biocompatibility, and the use of these materials with bioactivity is expected to promote optimal periapical healing.^3^^,^^4^ However, most traditional root canal filling materials were developed with a focus on their physical properties and they rarely possess bioactive properties.^2^^,^^5^ Hydraulic calcium silicate-based (HCSB) materials were introduced as endodontic materials with high bioactivity.^6^^,^^7^ The most commonly used HCSB material is mineral trioxide aggregate (MTA), which is formulated from Portland cement combined with bismuth oxide powder for radiopacity.^8^^,^^9^ MTA has shown favorable clinical outcomes for direct pulp capping, root-end filling, and iatrogenic root perforation repair,^8^ which may be attributed to its good biocompatibility,^10^ mineralized tissue-inductivity^10^ and anti-inflammatory properties.^11^ These biological properties of MTA are related to its continuous release of Ca^2+^.^12^

HCSB materials have been advocated for use as root canal sealers because of their good biocompatibility and bioactivity, and several premixed HCSB sealers formulated with calcium silicates as a major component have been developed.^13^ EndoSequence BC sealer (BC; Brasseler USA, Savannah, GA, USA) is the most studied premixed HCSB sealer and its composition includes calcium silicates, calcium phosphate monobasic, calcium hydroxide, filler, and thickening agents. BC promotes significant osteoblastic differentiation,^14^, ^15^, ^16^ and exhibits anti-inflammatory effects.^17^ Bio-C Sealer (BioC; Angelus, Londrina, PR, Brazil), a recently-developed HCSB sealer, contains tricalcium silicate, dicalcium silicate, and tricalcium aluminate as its major ingredients and has mineralization-inducing potential in human periodontal ligament stem cells (hPDLSCs).^18^ Well-Root ST (WST; Vericom, Gangwon-Do, Korea), another recently-developed HCSB sealer, contains calcium aluminosilicate compound as its major component and is reported to promote alkaline phosphatase activity, osteoblastic marker gene expression, and mineralized nodule formation in hPDLSCs.^19^

However, the mineralized tissue-inductive ability and anti-inflammatory properties of these recently-developed HCSB sealers have not been fully elucidated. Thus, this study aimed to compare the effects of three HCSB sealers, Bio-C sealer (BioC), Well-Root ST (WST), and EndoSequence BC sealer (BC), as well as AH Plus Jet (AHP; an epoxy resin-based sealer; Dentsply Sirona, York, PA, USA) as a control sealer, on osteoblastic differentiation/mineralization and proinflammatory mediator synthesis in macrophages.

## Materials and methods

### Cell culture

Kusa-A1 cells (p3-12, RCB2081; RIKEN BRC, Tsukuba, Japan), a bone marrow stromal cell line with osteoblastic properties derived from C3H/He mice,^20^^,^^21^ were maintained in alpha-modified minimum essential medium (α-MEM; FUJIFILM Wako Pure Chemical, Osaka, Japan) containing 10% fetal bovine serum (FBS; HyClone/GE Healthcare, Chicago, IL, USA) and penicillin-streptomycin-amphotericin B (FUJIFILM Wako Pure Chemical). RAW264.7 cells (p3-12, RCB0535; RIKEN BRC), a typical macrophage cell line,^22^ were cultured in Dulbecco's modified Eagle's medium (D-MEM; FUJIFILM Wako Pure Chemical) supplemented with heat-inactivated 10% FBS (HyClone/GE Healthcare) and penicillin-streptomycin-amphotericin B. The cultures were maintained at 37 °C with 5% CO_2_ and 100% humidity.

### Preparation of sealer extracts

BioC, WST, BC, and AHP (Table 1) were mixed following the manufacturer's instructions, used to fill polypropylene discs (3 mm height × 7.5 mm diameter), and incubated at 37 °C with 100% humidity for 2 days to achieve the set conditions. AHP, an epoxy resin-based sealer, was used as a control sealer against the three HCSB sealers in this study because AHP contains no typical bioactive components such as calcium silicates and calcium aluminosilicate. The set sealers were immersed in 3 mL of distilled water (DW) under shaking at room temperature for 24 h, and the sealer extracts were filtered (0.45-μm pore size, Sartorius, Göttingen, Germany) and mixed with α-MEM or D-MEM at a 1:4 ratio. Calcium chloride (CaCl_2_) was dissolved in DW at concentrations of 0, 5, 10, and 20 mM, and then mixed with α-MEM at a 1:4 ratio. DW was used as a negative control.

**Table 1:** Compositions of sealers.

Sealers	Lot No.	Composition
AH Plus Jet (AHP)	2110001419	Epoxy paste: diglycidil-bisphenol-A-ether, calcium tungstate, zirconium oxide, aerosol, dye. Amine paste: 1-adamantane amine, N,N′-dibenzyl-5-oxanonandiamine-1,9, TCD-diamine, calcium tungstate, zirconium oxide, aerosol, silicone oil.
Bio-C sealer (BioC)	59965	Tricalcium silicate, dicalcium silicate, tricalcium aluminate, calcium oxide, zirconium oxide, silicon dioxide, polyethylene glycol, iron oxide.
Well-Root ST (WST)	WR160100	Calcium aluminosilicate compound, zirconium oxide, filler, thickening agents.
EndoSequence BC sealer (BC)	(10)21003SP	Zirconium oxide, calcium silicates, calcium phosphate monobasic, calcium hydroxide, filler, thickening agents.

### Cell proliferation assay

Kusa-A1 cells (1.5 × 10^3^ cells/well) were seeded in 96-well plates and cultured for 24 h followed by the application of sealer extracts or CaCl_2_ for 24, 48, and 72 h. Cell proliferation was measured by WST-8 Assay (Cell Counting Kit-8; Dojindo, Kumamoto, Japan) following the manufacturer's instructions. The optical density at 450 nm (OD450) was measured using a 96-well plate reader (Sunrise, Tecan, Männedorf, Switzerland).

### Mineralized nodule formation

Kusa-A1 cells (2 × 10^4^ cells/well) were seeded in 48-well plates and cultured in α-MEM containing 10% FBS for 24 h. Then, the medium was replaced with a mineralization medium containing β-glycerophosphate (5 mM; Merck, Rahway, NJ, USA), l-ascorbic acid (0.2 mM; FUJIFILM Wako Pure Chemical), and dexamethasone (1 nM; FUJIFILM Wako Pure Chemical), with or without sealer extracts or CaCl_2_. Mineralized nodules were stained with Alizarin red S (FUJIFILM Wako Pure Chemical) on days 3 and 6. For quantification, images were taken and the number of pixels in a region of interest was quantified using ImageJ software (ver. 1.53; National Institutes of Health, Bethesda, MD, USA).

### Reverse transcription-quantitative polymerase chain reaction

Kusa-A1 cells (5 × 10^4^ cells/well) were seeded in 24-well plates and cultured without sealer extracts for 24 h followed by the application of sealer extracts or CaCl_2_ for 24 h. RAW264.7 cells (1 × 10^5^ cells/well) were seeded in 12-well plates and cultured in D-MEM containing 10% FBS for 24 h followed by serum starvation for 24 h. Then, RAW264.7 cells were stimulated with lipopolysaccharide (LPS, 100 ng/mL, *Escherichia coli* O111: B4, Merck) with or without sealer extracts for 3 h. Total RNA was extracted with a QuickGene-RNA extraction kit (FUJIFILM Wako Pure Chemical) and converted into cDNA with a PrimeScript cDNA synthesis kit (Takara Bio, Kusatsu, Japan). Quantitative polymerase chain reaction (qPCR) was performed with GoTaq qPCR MasterMix (Promega, Madison, WI, USA) and specific primers (Table 2) using the CFX96 real-time PCR detection system (Bio-Rad, Hercules, CA, USA). Relative normalized expression of the target genes was calculated following the ΔΔCt method against the housekeeping gene, β-actin.

**Table 2:** Primer sequences.

Gene	Forward Primer (5′-3′)	Reverse Primer (5′-3′)	Accession No.	size (bp)
IL-1α	CACCTTACACCTACCAGAGTGATTT	ATTTAACCAAGTGGTGCTGAGATA	NM_010554	137
IL-1β	AAACGGTTTGTCTTCAACAAGATAG	AATTATGTCCTGACCACTGTTGTTT	NM_008361	141
IL-6	TGGATGCTACCAAACTGGATATAAT	TCTGGCTTTGTCTTTCTTGTTATCT	NM_031168	130
TNF-α	GATGGGTTGTACCTTGTCTACTCC	GAGGTTGACTTTCTCCTGGTATGAG	NM_013693	120
Oc	AGGGCAATAAGGTAGTGAACAGAC	CATACTGGTCTGATAGCTCGTCAC	NM_007541	129
Opn	GATGTGATCGATAGTCAAGCAAGTT	TTCGGAATTTCAGATACCTATCATC	NM_001204201	130
β-actin	AATGATCTTGATCTTCATGGTGCTA	GTAAAGACCTCTATGCCAACACAGT	NM_007393	122

IL: interleukin, Oc: osteocalcin, Opn: osteopontin, TNF: tumor necrosis factor.

### Enzyme-linked immunosorbent assays

RAW264.7 cells (1 × 10^5^ cells/well) were seeded in 12-well plates and cultured in D-MEM containing 10% FBS for 24 h followed by serum starvation for 24 h. Then, RAW264.7 cells were stimulated with LPS (100 ng/mL) with or without sealer extracts for 24 h. Proinflammatory cytokines in the culture medium were measured by enzyme-linked immunosorbent assay kits (interleukin [IL]-6 and tumor necrosis factor [TNF]-α DuoSet, R&D Systems, Minneapolis, MN, USA) following the manufacturer's protocol. 3,3′,5,5′-tetramethylbenzidine (SureBlue TMB Microwell Peroxidase Substrate, KPL, Milford, MA, USA) and sulfuric acid (0.6 N) were used as a chromogenic substrate and a reaction stop solution, respectively. Color intensity was measured with a microplate reader (Sunrise) at OD450.

### Scanning electron microscopy

Mixed sealers used to fill in polypropylene discs (2 mm height × 9 mm diameter) were allowed to set for 2 days followed by rinsing with DW for 24 h, sterilization with 70% ethanol for 15 min, and washing with phosphate-buffered saline for 5 min. Then, samples were placed in a 24-well plate with 2 mL α-MEM for 24 h, and Kusa-A1 cells (2.5 × 10^4^ cells/well) were seeded on the samples and cultured for 72 h. Following fixation in 2.5% glutaraldehyde (FUJIFILM Wako Pure Chemical), the samples were dehydrated, dried in a critical-point dryer (HCP 2, Hitachi, Tokyo, Japan), sputter-coated with platinum using an ion-coater (E102, Hitachi), and scanned with an electron microscope (JSM-7900, JEOL, Japan) at an accelerating voltage of 15 kV.

### Ca^2+^ concentration measurement

Ca^2+^ concentration in the sealer extracts was measured by inductively coupled plasma atomic emission spectrometry (IC-7000 ver. 2; Shimadzu, Kyoto, Japan).

### Statistical analysis

GraphPad Prism (ver.9; GraphPad, La Jolla, CA, USA) was used for statistical analyses. Following the verification of data normality and variance homogeneity using the Shapiro-Wilk test and Levene's test, respectively, data were analyzed using one-way analysis of variance followed by the Tukey-Kramer test. A *P*-value less than 0.05 was considered statistically significant.

## Results

### Effects of sealer extracts on osteogenic differentiation and mineralization in Kusa-A1 cells

**Fig. 1:**
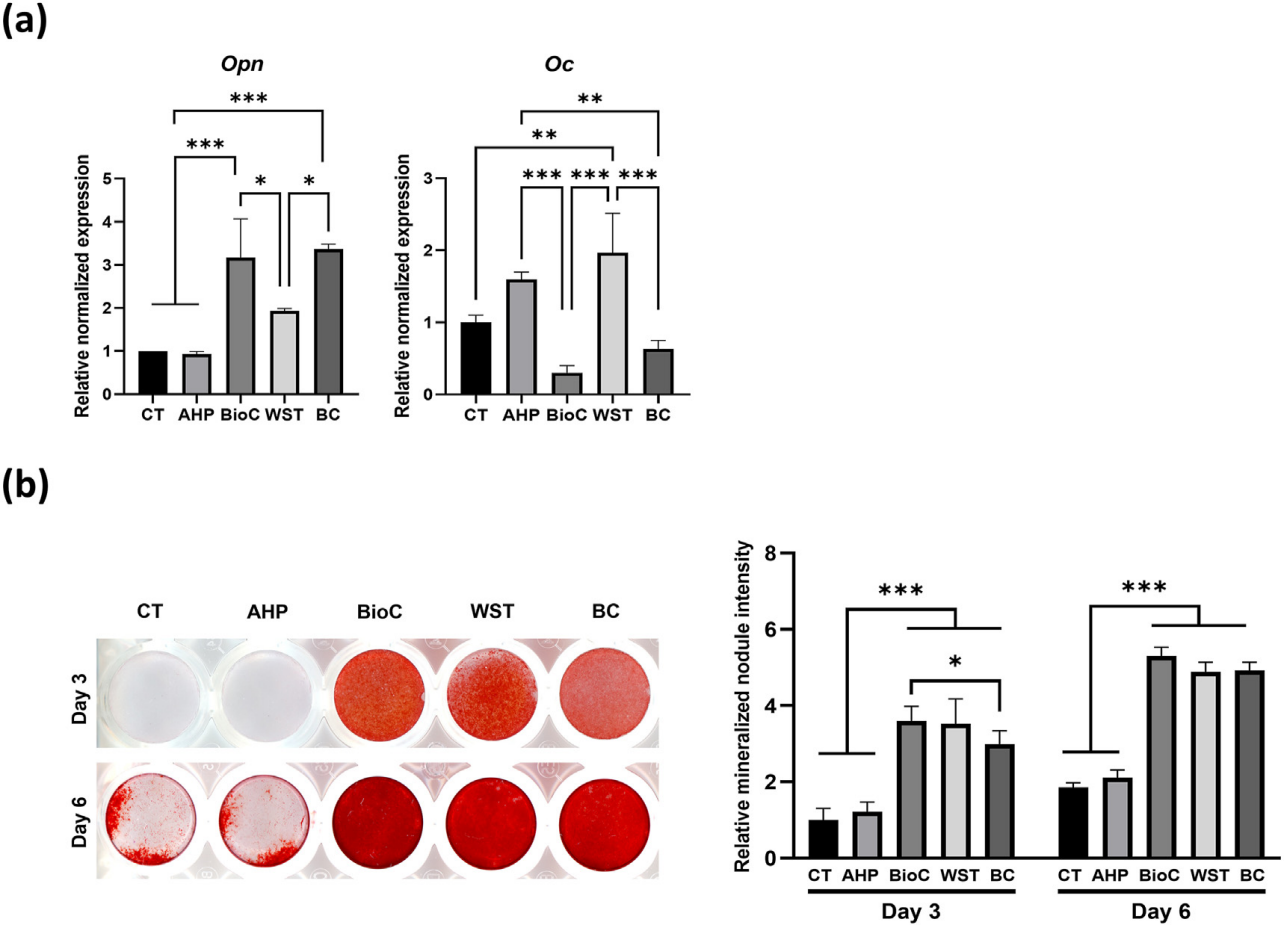
Effect of BioC, WST, BC, and AHP on osteogenic gene expression (a) and mineralized nodule formation (b) in Kusa-A1 cells. **(a)** mRNA expression of osteopontin (Opn) was significantly upregulated by BioC and BC, and mRNA expression of osteocalcin (Oc) was significantly upregulated by WST in Kusa-A1 cells. AHP had no effect on the mRNA expressions of Opn and Oc. Data are shown as the mean and SD (n = 3). **(b)** Mineralized nodule formation of Kusa-A1 cells was significantly increased by BioC, WST, and BC. AHP had no effect on mineralized nodule formation. Data are shown as a ratio relative to the negative control (distilled water) at day 3 (mean and SD, n = 5). AHP: AH Plus Jet, BC: EndoSequence BC sealer, BioC: Bio-C sealer, CT: negative control, WST: Well-Root ST. ∗*P* < 0.05, ∗∗*P* < 0.01, ∗∗∗*P* < 0.001.

We first evaluated the mRNA expression of osteopontin (Opn) and osteocalcin (Oc), which are typical osteoblastic markers,^23^^,^^24^ especially Oc is reported to be involved in mineralization.^24^ mRNA expression of Opn was significantly upregulated by BioC and BC in Kusa-A1 cells compared with the negative control (DW) and AHP (*P* < 0.001, Fig. 1a), and the mRNA expression of Oc was significantly upregulated by WST in Kusa-A1 cells compared with the negative control (*P* < 0.01, Fig. 1a). AHP failed to promote the mRNA expressions of Opn and Oc (Fig. 1a). Clear mineralized nodules were detected in Kusa-A1 cells approximately 6 days after culture in mineralization medium (Fig. 1b). The application of BioC, WST, and BC promoted significant Alizarin red S staining at 3 and 6 days compared with the negative control (DW) and AHP (*P* < 0.001, Fig. 1b).

### Effects of sealer extracts on Kusa-A1 cell proliferation

**Fig. 2:**
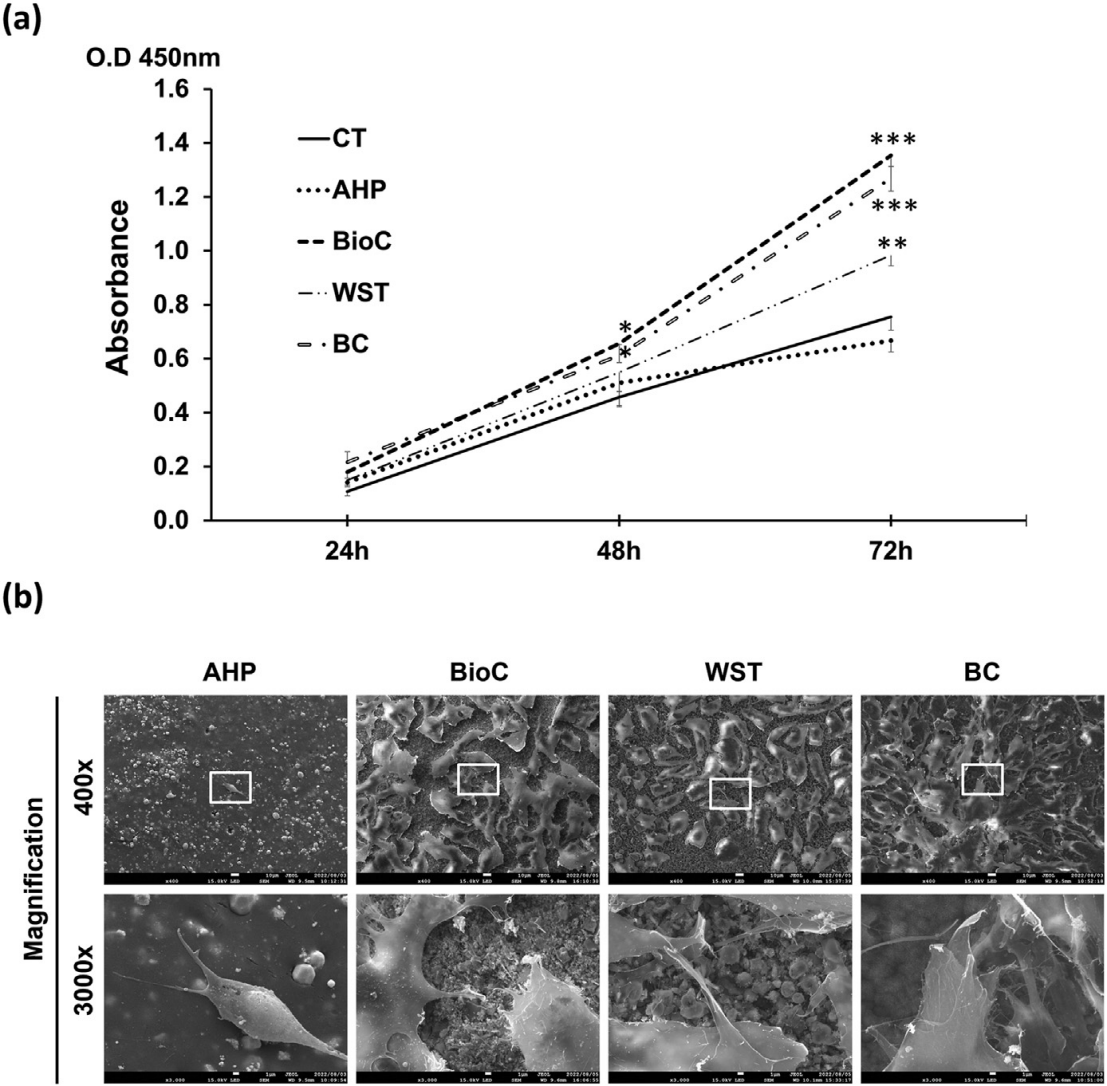
**(a)** Proliferation of Kusa-A1 cells was significantly increased by BioC and BC at 48 and 72 h. WST significantly upregulated the proliferation of Kusa-A1 cells at 72 h. AHP had no effect on the proliferation of Kusa-A1 cells. Data are shown as the mean and SD (n = 4). **(b)** Well-attached Kusa-A1 cells were observed on set BioC, WST, and BC, but attached cells were rarely observed on AHP. Representative images from three separate experiments are shown. AHP: AH Plus Jet, BC: EndoSequence BC sealer, BioC: Bio-C sealer, CT: negative control (distilled water), WST: Well-Root ST. ∗*P* < 0.05, ∗∗*P* < 0.01, ∗∗∗*P* < 0.001.

The proliferation of Kusa-A1 cells was increased significantly by BioC and BC at 48 and 72 h (*P* < 0.05 and 0.001, respectively; Fig. 2a) and by WST at 72 h (*P* < 0.01, Fig. 2) compared with the negative control (DW) and AHP. BioC and BC promoted significant cell proliferation compared with WST at 72 h (*P* < 0.01, Fig. 2a).

### Attachment of Kusa-A1 cells to set sealers

Kusa-A1 cells cultured on set BioC, WST, and BC were well-spread and attached to the sealers by numerous filopodia or pseudopodia. However, well-attached cells were rarely observed on AHP at 72 h of cell culture (Fig. 2b).

### Effects of Ca^2+^ on osteogenic differentiation/mineralization and proliferation of Kusa-A1 cells

**Fig. 3:**
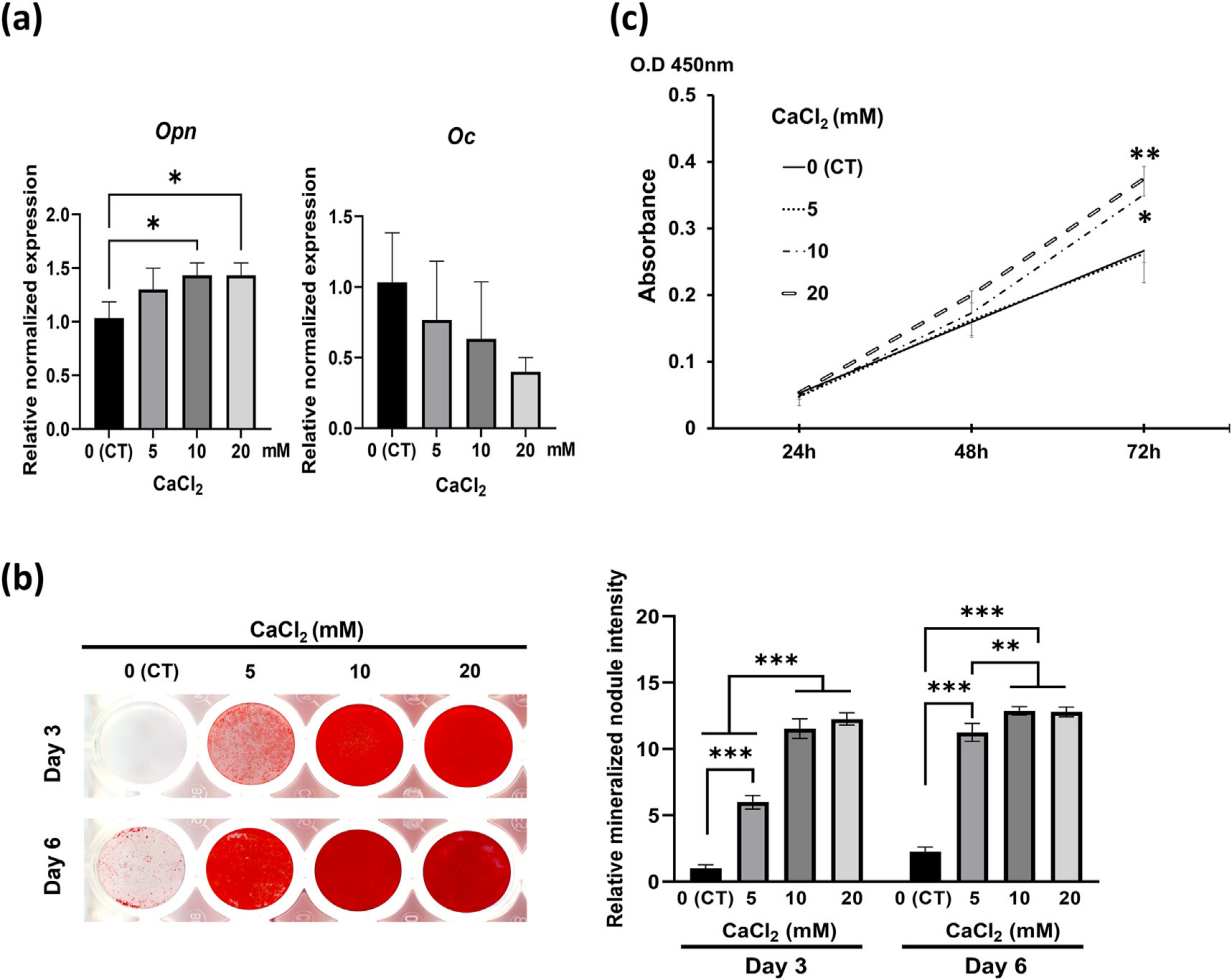
Effects of Ca^2+^ on osteogenic gene expression (a), mineralized nodule formation (b), and proliferation (c) of Kusa-A1 cells. **(a)** mRNA expression of Opn was significantly upregulated by 10 and 20 mM CaCl_2_. Data are shown as the mean and SD (n = 3). **(b)** Mineralized nodule formation of Kusa-A1 cells was significantly increased by 5, 10, and 20 mM CaCl_2_. Data are shown as a ratio relative to the negative control (distilled water) at day 3 (mean and SD, n = 4). **(c)** Proliferation of Kusa-A1 cells was significantly increased by 10 and 20 mM CaCl_2_ at 72 h. Data are shown as the mean and SD (n = 4). CT: negative control. ∗*P* < 0.05, ∗∗*P* < 0.01, ∗∗∗*P* < 0.001.

The mean amount of Ca^2+^ released from BioC, WST, and BC was 917, 293, and 990 mg/L, respectively (Table 3). The mRNA expression of Opn in Kusa-A1 cells was increased significantly by 10 and 20 mM CaCl_2_ compared with the negative control (DW) (*P* < 0.05, Fig. 3a). The mRNA expression of Oc was not affected by CaCl_2_ (Fig. 3a). Mineralized nodule formation was enhanced in Kusa-A1 cells by CaCl_2_ at 3 days (Fig. 3b), and the intensity of Alizarin red S staining related to CaCl_2_ application was significant at 3 and 6 days compared with the negative control (*P* < 0.001, Fig. 3b). Ten and 20 mM CaCl_2_ significantly upregulated the proliferation of Kusa-A1 cells compared with the negative control at 72 h (*P* < 0.05 and 0.01, respectively; Fig. 3c).

**Table 3:** Ca^2+^ concentration in the sealer extracts.

Samples	Ca^2+^ (mg/L)
Distilled water	0
AHP	13 ± 12
BioC	917 ± 46
WST	293 ± 45
BC	990 ± 58

Data are shown as the mean ± SD (n = 3). AHP: AH Plus Jet, BC: EndoSequence BC sealer, BioC: Bio-C sealer, WST: Well-Root ST.

### Effects of sealer extracts on proinflammatory cytokine synthesis in RAW264.7 cells

**Fig. 4:**
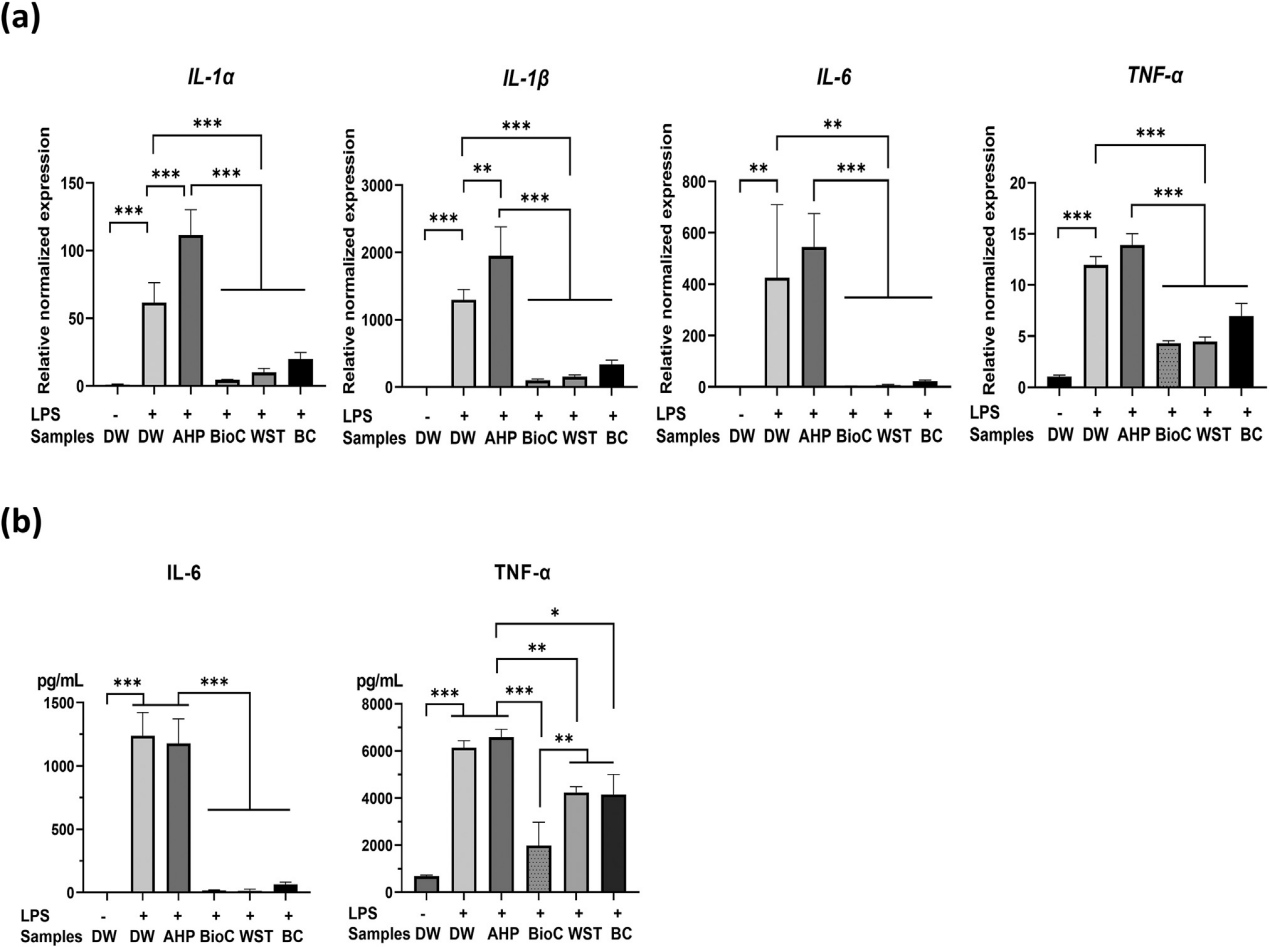
Effects of BioC, WST, BC, and AHP on proinflammatory mediator synthesis in LPS-stimulated RAW264.7 cells. **(a)** mRNA expressions of IL-1α, IL-1β, IL-6, and TNF-α were significantly upregulated by LPS stimulation and significantly downregulated by BioC, WST, and BC in RAW264.7 cells. AHP significantly enhanced the mRNA expressions of IL-1α, IL-1β, IL-6, and TNF-α in LPS-stimulated RAW264.7 cells. Data are shown as the mean and SD (n = 3). **(b)** Syntheses of IL-6 and TNF-α were significantly upregulated by LPS stimulation and significantly downregulated by BioC, WST, and BC in RAW264.7 cells. AHP had no effect on the synthesis of IL-6 and TNF-α in LPS-stimulated RAW264.7 cells. Data are shown as the mean and SD (n = 3). AHP: AH Plus Jet, BC: EndoSequence BC sealer, BioC: Bio-C sealer, DW: distilled water, WST: Well-Root ST. ∗*P* < 0.05, ∗∗*P* < 0.01, ∗∗∗*P* < 0.001.

The mRNA expressions of IL-1α, IL-1β, IL-6, and TNF-α and protein levels of IL-6 and TNF-α were upregulated significantly in LPS-stimulated RAW264.7 cells (*P* < 0.001 or 0.01; Fig. 4a and b). BioC, WST, and BC significantly downregulated the mRNA expressions of IL-1α, IL-1β, IL-6, and TNF-α (*P* < 0.001 or 0.01) and protein levels of IL-6 (*P* < 0.001) and TNF-α (*P* < 0.001, 0.01, and 0.05, respectively) in LPS-stimulated RAW264.7 cells (Fig. 4a and b). On the contrary, AHP failed to downregulate the mRNA expressions of IL-1α, IL-1β, IL-6, and TNF-α and protein levels of IL-6 and TNF-α in LPS-stimulated RAW264.7 cells (Fig. 4a and b).

## Discussion

The current study revealed that all the tested HCSB materials but not AHP promoted osteoblastic proliferation and differentiation/mineralization in Kusa-A1 cells. The three HCSB materials released relatively high amounts of Ca^2+^ while AHP rarely released it (Table 3), indicating that Ca^2+^ is responsible for these properties. This was supported by the finding that 10 and 20 mM CaCl_2_ promoted the proliferation and differentiation/mineralization of Kusa-A1 cells (Fig. 3). The Ca^2+^ concentrations of 4-times diluted 10 and 20 mM CaCl_2_ were almost equivalent to 100 and 200 mg/L, respectively, which are approximate to those in the 4-times diluted extracts of BioC, WST, or BC (229, 73, and 248 mg/L, respectively). Ca^2+^ at 2–4 mM (80–160 mg/L) is suitable for the proliferation and survival of osteoblasts, and 6–8 mM (240–320 mg/L) Ca^2+^ favors osteoblast differentiation and matrix mineralization.^25^ Because α-MEM contains 72 mg/L Ca^2+^, final Ca^2+^ concentrations in the culture medium containing 4-times diluted BioC, WST, or BC extracts approximated 301, 145, and 320 mg/L, respectively. Therefore, promotion of the proliferation and differentiation/mineralization of Kusa-A1 cells induced by the HCSB sealer extracts may be associated with Ca^2+^ release from the HCSB sealers, although factors other than Ca^2+^ might also be involved. BioC and BC upregulated the mRNA expression of Opn (Fig. 1a), a typical osteoblastic marker,^23^ which was also induced by CaCl_2_ (10 and 20 mM, Fig. 3a), suggesting that Ca^2+^ released from BioC and BC are involved in the upregulation of Opn mRNA expression. In contrast, the mRNA expression of Oc, an osteoblastic marker closely related to mineralization,^24^ was promoted by WST (Fig. 1a) but not by CaCl_2_ (Fig. 3a). Thus, the upregulation of Oc mRNA may involve factor(s) other than Ca^2+^ released from WST. The three HCSB sealers may release different concentrations of aluminum and/or silicon, which were reported to be inhibitory^26^ and stimulatory,^27^ respectively, to osteoblastic differentiation. Further analysis is required to determine whether and how aluminum and silicon are involved in the mineralized tissue-inductive ability of these materials.

This study demonstrated that the marked downregulation of proinflammatory cytokine synthesis was induced by the application of all three HCSB sealer extracts to LPS-stimulated RAW264.7 cells (Fig. 4). IL-1α, IL-1β, and TNF-α are essential factors for osteoclastogenesis and pathogenesis in periapical lesions.^28^ TNF-α regulates receptor activator NF-κB ligand expression, which is an essential signaling factor that induces osteoclast formation^29^ via IL-1 that directly stimulates the differentiation of osteoclast precursors.^30^ IL-6 has pathological effects on chronic inflammation.^31^ Therefore, the downregulation of these proinflammatory mediators might contribute to the healing of periapical lesions accompanied by bone regeneration. The three HCSB sealers released high concentrations of Ca^2+^ (Table 3), which may contribute to their anti-inflammatory effects. On the contrary, AHP, which rarely released Ca^2+^ (Table 3), failed to induce the anti-inflammatory effects in LPS-stimulated RAW264.7 cells (Fig. 4). Ca^2+^ released from MTA was reported to induce Ca^2+^ influx via the calcium-sensing receptor (CaSR), and activate calcineurin/nuclear factor of activated T-cells (NFAT) signaling in RAW267.4 cells.^11^ CaSR/NFAT signaling then induces early growth response 2, which inhibits the mRNA expressions of IL-1α and IL-6, and promotes the expression of IL-10, a typical anti-inflammatory cytokine,^32^ and suppressor of cytokine signal 3, a major regulator of inflammation.^33^ In addition, Ca^2+^ released from MTA stimulates CaSR/NFAT signaling, which further stimulates the osteogenic differentiation of human dental pulp cells.^34^ It is tempting to speculate that the activation of CaSR/NFAT signaling is also involved in the osteoblastic differentiation/mineralization of Kusa-A1 cells induced by the three HCSB sealers.

Good biocompatibility is an essential property for endodontic sealers; however, a direct comparison of biocompatibility between BioC, WST, and BC has not been reported. This study demonstrated that the three sealers did not inhibit cell growth and that Kusa-A1 cells attached well to set BioC, WST, and BC by numerous filopodia/pseudopodia (Fig. 2b). These findings support the low cytotoxicity and good biocompatibility reported for these HCSB sealers.^15^^,^^16^^,^^18^^,^^35^, ^36^, ^37^, ^38^^,^^40^^,^^41^ BioC has lower toxicity than AHP in hPDLSCs^18^ and exhibits similar toxicity to that of BC in a lethality assay involving brine shrimp, *Artemia salina*.^35^ Furthermore, subcutaneously implanted BioC induces lower infiltration of inflammatory cells and IL-6 synthesis compared to AHP.^36^ WST has been reported to be more biocompatible and less cytotoxic than AHP in human dental pulp stem cells,^37^ human tooth germ stem cells,^37^ hPDLSCs,^37^^,^^38^ and MC3T3-E1, a typical osteoblastic cell line.^39^^,^^40^ BC also shows good biological properties related to hPDLSCs^16^^,^^41^ and a murine osteoblast precursor cell line, IDG-SW3 compared with AHP.^15^ Here, we showed that BioC, WST, and BC were equally biocompatible and less toxic compared with AHP.

In conclusion, the three HCSB sealers, BioC, WST, and BC exhibited high biocompatibility with Kusa-A1 cells and promoted their osteoblastic differentiation/mineralization probably by the release of Ca^2+^. Moreover, BioC, WST, and BC reduced the synthesis of proinflammatory cytokines IL-1α, IL-1β, IL-6, and TNF-α from LPS-stimulated RAW264.7 cells. These properties are considered beneficial for the healing of periapical lesions accompanied by new alveolar bone formation and apical closure with newly-formed mineralized tissue(s). Our data provide basic evidence supporting the notion that these HCSB sealers possess suitable biological properties for use as root canal filling materials.

## Declaration of competing interest

The authors declare that they have no known competing financial interests or personal relationships that could have appeared to influence the work reported in this paper.
